# The Translationally Controlled Tumor Protein (TCTP), a Novel Antigen of *Babesia bovis*, Participates in the Establishment of Acute Infection and Contains Neutralizing B-Cell Epitopes

**DOI:** 10.3390/pathogens14050502

**Published:** 2025-05-20

**Authors:** Chyntia Pérez-Almeida, Diego Josimar Hernández-Silva, Edwin Esaú Hernández-Arvizu, Masahito Asada, Shin-ichiro Kawazu, Massaro W. Ueti, José Guadalupe Gomez-Soto, Urso Martín Dávila-Montero, Carlos A. Vega y Murguía, Juan Mosqueda

**Affiliations:** 1 Immunology and Vaccine Research Laboratory, Natural Sciences College, Autonomous University of Queretaro, Queretaro 76230, Mexico; chyntiaq@gmail.com (C.P.-A.); diego.hernandez@uaq.mx (D.J.H.-S.); esau.hernandez@uaq.mx (E.E.H.-A.); 2 Faculty of Veterinary Medicine and Animal Husbandry, National Autonomous University of México, Colony C.U., Coyoacán, Mexico City 04510, Mexico; 3 National Research Center for Protozoan Diseases, Obihiro University of Agriculture and Veterinary Medicine, Inadacho, Nishi 2-13, Obihiro 080-8555, Hokkaido, Japan; masada@obihiro.ac.jp (M.A.); skawazu@obihiro.ac.jp (S.-i.K.); 4 Animal Diseases Research Unit, Agricultural Research Service, US Department of Agriculture, Pullman, WA 99164, USA; massaro.ueti@usda.gov; 5 Natural Sciences College, Autonomous University of Queretaro, Queretaro 76230, Mexico; jose.gomez@uaq.mx (J.G.G.-S.); ursodavila@uaq.mx (U.M.D.-M.); vegaymurguia.carlosagustin@gmail.com (C.A.V.y.M.)

**Keywords:** translationally controlled tumor protein, bovine babesiosis, *Babesia bovis*

## Abstract

*Babesia bovis* is a protozoan parasite that causes babesiosis in cattle. It has been hypothesized that in apicomplexan parasites, translationally controlled tumor protein (TCTP) interferes with the host immune response by inhibiting B cell proliferation. The aim of this study was the characterization of *B. bovis* TCTP (BboTCTP) and the evaluation of its expression, immunogenicity and role in infection. The *tctp* gene was identified and sequenced from *B. bovis* isolates and revealed a high conservation. Expression was confirmed in intraerythrocytic stages by Western blot and confocal microscopy. Synthetic peptides containing predicted B cell epitopes were used to immunize cattle, followed by a challenge with a virulent *B. bovis* strain. Immunized animals showed milder clinical signs and faster recovery compared to controls. Sera from non-immunized animals exhibited lower total IgG levels after challenge (*p* < 0.05), while sera from immunized animals induced significant in vitro invasion inhibition (32–33%). These results suggest that BboTCTP is immunogenic and may play a role in modulating the host immune response. The results provide novel insights into *B. bovis* biology and support BboTCTP as a promising candidate for further evaluation as a vaccine antigen. Future studies should explore its immunomodulatory mechanisms and potential use in combined vaccine formulations.

## 1. Introduction

Bovine babesiosis is a disease caused by parasites of the genus *Babesia*. It has a worldwide distribution and is transmitted by different species of ticks, depending on the climatic conditions [1]. The most common and economically relevant species that infect cattle are *Babesia bovis*, *B. bigemina* and *B. divergens* [2,3]. *B. bovis* has the greatest impact on cattle due to its high virulence and high mortality rate compared to other species [4]. Although babesiosis is a treatable disease, the diagnosis is often inaccurate due to the lack of specific signs, resulting in a high economic impact in endemic areas. By better understanding the pathogenic mechanisms by which *Babesia* damages the organism, efficient strategies for controlling babesiosis can be developed.

The translationally controlled tumor protein (TCTP) is a highly conserved protein that is expressed in eukaryotic organisms. TCTP expression is regulated at both the transcriptional and translational levels and by a variety of extracellular signals. TCTP has been implicated in important cellular processes such as cell growth, cell cycle progression, malignant transformation and the protection of cells against various stresses and apoptosis. In addition, TCTP has been shown to have an extracellular function similar to that of cytokines [5].

TCTP has been studied in various Apicomplexa protozoa. It has been demonstrated that this protein is secreted into the host serum in concentrations directly correlated with the percentage of parasitemia, postulating the involvement of this protein in the establishment of infection by interfering with the host immune response [6,7,8,9]. It is not known if this protein is secreted by parasites of the phylum Apicomplexa through exosomes as it occurs in mammals [10]. It has been demonstrated that *Plasmodium* TCTP interacts with B cells, eosinophils and basophils, in addition to binding to artemisinin and calcium [6,11,12]. In vitro, this protein was shown to induce histamine secretion in basophils and IL-8 secretion in eosinophils, but with less intensity than human TCTP [7]. It has also been proposed that *Plasmodium falciparum* TCTP inhibits B-cell proliferation [13] and inhibits IL-6 secretion in vivo [9]. These studies have led to the postulate that *Plasmodium* TCTP competes with human TCTP for receptors on cells, interfering with the functions of human TCTP [12].

This protein is structurally similar between different taxonomic groups, however, differences in the three-dimensional structure of the protein have been described in species of the phylum Apicomplexa, maintaining these structural characteristics within the phylum [14,15,16]. The main similarity of this protein among organisms of the phylum Apicomplexa is in the receptor binding domain, where these organisms have an additional alpha helix instead of the β-sheet present in other taxonomic groups. This helix is formed between amino acids 22 to 30, generating a conformation with higher affinity to the receptor compared to the protein in mammals [13,17]. In addition, *Plasmodium* TCTP contains a transduction domain located at the NH_2_-terminal end, which might allow a more efficient internalization of the protein into the cell than mammalian TCTP [13]. These structural differences could allow TCTP to block the interaction of host TCTP with its receptor on B lymphocytes, preventing their activation and proliferation and thus the activation of the immune response. This is probably related to the decrease in memory B-cells reported in malaria [13,16,17]. Despite these insights from *Plasmodium*, the function of TCTP in *Babesia bovis* remains unknown. To date, no studies have characterized TCTP in *B. bovis* or explored its potential role in host–parasite interactions. In view of the structural differences described in apicomplexan TCTPs and their proposed immunomodulatory effects, investigation of BboTCTP may reveal novel mechanisms of immune evasion and identify new vaccine targets. The aim of this study was to characterize TCTP of *B. bovis* and to evaluate its involvement in the establishment of infection.

## 2. Materials and Methods

### 2.1. Identification of tctp Gene in the Genome of B. bovis

The *tctp* gene was identified in the *B. bovis* genome by performing a BLAST analysis through amino acid sequence homology using the National Center for Biotechnology Information (NCBI) Protein database (https://blast.ncbi.nlm.nih.gov, accessed on 13 September 2018) using as a query the *Plasmodium falciparum* TCTP sequence (GenBank accession no. 3P3K_A).

### 2.2. DNA Extraction, Amplification and Sequencing of tctp Gene

DNA extraction was performed from different samples of ticks and infected blood with *B. bovis*. The DNA extraction from ticks was performed with DNeasy Blood& Tissue kit (No. 69506, Qiagen, Hilden, Germany) following the purification of total DNA from arthropods using the DNeasy Blood & tissue kit according to the manufacturer’s instruction. The eluate was stored at −20 °C. The DNA extraction from blood infected with *B. bovis* was performed with the same kit according to the manufacturer’s instruction. To analyze if the sequence of *tctp* is conserved among different isolates—Michoacán-México isolate, Zacatecas-México isolate, Nayarit-México isolate, Puebla-México isolate, Jalisco-México isolate, Trinidad and Tobago isolate, and the reference strain T2Bo—the *tctp* gene of each strain was amplified by PCR using oligonucleotides BbotctpF (5’-TGA TGT CAC AAG GGA TGT GC-3’) and BbotctpR (5′-TCC AAG TCA ACA GGA AGA TTG-3′), which amplify an amplicon of 1199 bp. The PCR conditions were initially denaturing at 95 °C for 120 s min, followed by 35 cycles at 95 °C for 30 s, 56 °C for 30 s and 72 °C for 60 s, with a final extension at 72 °C for 420 s, with PCR Master Mix (Promega, Madison, WI, USA). The amplicons were cloned into a TOPO TA cloning^®^ (Invitrogen, Carlsbad, CA, USA) according to the manufacturer’s instructions, and plasmids were extracted using the Illustra™ plasmidPred Mini Spin^®^ kit (28-9042), (GE Healthcare^®^, Chicago, IL, USA). The plasmids were sequenced by the UNAM Institute of Biotechnology (IBT) in Morelos, Mexico.

### 2.3. Transcription Analysis by RT-PCR

Total RNA from *B. bovis*-infected erythrocytes was purified using Trizol (Invitrogen, Carlsbad, CA, USA). Then, 1 µg of total RNA was reverse transcribed using Tetro cDNA synthesis kit (Bioline, London, UK) according to the manufacturer’s instructions. An amount of 2 µL of cDNA from each sample and Master Mix 2× (Bioline, London, UK) was used for PCR. The oligonucleotides were pF (5′-CAG TAA GAG CCT CCT TAA TG-3′) and pR (5′-CAA GCG TTG CCT TTG AAG TT-3′). They amplify a 426 bp amplicon of the *tctp* gene. The amplification conditions were the following: an initial denaturation step at 95 °C for 60 s, followed by 30 cycles at 95 °C for 15 s, 61.9 °C for 15 s, and 72 °C for 30 s, with a final extension step at 72 °C for 300 s.

### 2.4. TCTP Topology Analysis

The structure of *B. bovis* TCTP was predicted and compared. The sequences obtained were aligned with the bioinformatics tool Clustal Omega (available at www.ebi.ac.uk/Tools/msa/clustalo/, accessed on 8 October 2018) [18]. A consensus sequence was obtained and with this the topology of the protein was analyzed using different bioinformatics tools. SignalP-5.0 (available at https://services.healthtech.dtu.dk/service.php?SignalP-5.0, accessed on 24 June 2019) [19] was used to predict the presence of a signal peptide. TMHMM server 2.0 (available at https://services.healthtech.dtu.dk/service.php?TMHMM-2.0, accessed on 24 June 2019) [20] predicted the presence transmembrane regions, and Pfam (available at http://pfam.xfam.org/, accessed on 24 June 2019) [21] identified protein domains. Multiple alignments were also performed using Clustal Omega [18] to identify the similarity percentage with TCTP from other species.

### 2.5. Prediction of the Three-Dimensional Structure of B. bovis TCTP

The three-dimensional structure of *B. bovis* TCTP was predicted using the SWISS-MODEL protein homology modeling tool (available at https://swissmodel.expasy.org/, accessed on 14 April 2020) [22]. The structure prediction was performed using the previously obtained amino acid sequence of BboTCTP. This tool identified the three-dimensional structure of *P. falciparum* TCTP (PDB accession number: 3P3K) and used it as a template [11]. A homology-based structural model was generated, and the quality of the model was evaluated using the Global Model Quality Estimation (GMQE) and Qualitative Model Energy Analysis (QMEAN) scores provided by SWISS-MODEL. The structural visualization provided by the server allowed the identification and highlighting of secondary structure elements such as alpha-helices and beta-sheets.

### 2.6. Prediction of Peptides Containing B-Cell Epitopes

Subsequently, B-cell epitope predictions in the conserved peptides were carried out using BCEpred (available at https://webs.iiitd.edu.in/raghava/bcepred/index.html, accessed on 18 December 2019) [23], Bepipred 2.0 (available at https://services.healthtech.dtu.dk/services/BepiPred-2.0/, accessed on 18 December 2019) [24] and the Immune Epitope database (available at https://www.iedb.org/, accessed on 18 December 2019) [25].

### 2.7. Expression of TCTP in Babesia bovis

Western blot (WB) analysis was performed to evaluate the expression of TCTP. For this, *B. bovis*-infected red blood cells (iRBC) from culture were washed with saponin buffer (0.15%) saponin in PBS containing protease inhibitor (Protease Inhibitor Cocktail EDTA Free, ab270055, Abcam, Cambridge, UK) in ice for 600 s, mixed 3–4 times. The iRBC were then centrifuged at 5000× *g*/300 s. The supernatant was removed, and the pellet was washed 3 times with PBS containing protease inhibitor. The pellet was gently resuspended in a buffer containing SDS 2% + Triton 100× 1% in PBS with protease inhibitors (100 µL/ 200 µL iRBC, gently mixed). The sample was frozen (−80 °C/180 s) and thawed (room temperature) 3 times. The same protocols were used with uninfected erythrocytes. The sample was mixed with Laemmli buffer 2× (glycerol 20%, Tris HCl pH 6.8 120 mM, SDS 4%, bromophenol blue 0.02%, β-mercaptoethanol 2.5%) and boiled at 96 °C for 300 s. Proteins were separated on 15% SDS-polycrilamide gels and electrotransferred to nitrocellulose membranes for 1 h at 70 volts. The membrane was washed with TBS for 300 s and blocked with TBS containing 5% skim milk (Svelty®, Nestlé Japan Ltd., Tokyo, Japan) for 2 h at room temperature. The bovine anti-TCTP antiserum was obtained by immunizing cattle with a recombinant BboTCTP protein, which was expressed in *E. coli* and purified by His-tag affinity chromatography (Unpublished data). Bovine anti-TCTP antiserum was diluted 1:5000 in TBS with 1% skim milk and incubated with the membrane overnight at 4 °C. The membrane was washed twice with TBS. The membrane was incubated with peroxidase affinity goat anti-bovine IgG H + L (cod. 6030 101-035-165, Jackson ImmunoResearch, Baltimore, MD, USA) diluted 1:15,000 in TBS for 1 h with agitation at room temperature and washed twice with TBS. Proteins of interest were detected by chemiluminescence using ECL-Prime Western Blotting Detection Reagent (GE Healthcare, Chicago, IL, USA) and the reaction was recorded in a ChemiDoc^TM^ Imaging System, Biorad (Hercules, CA, USA).

### 2.8. Generation of Specific Antibodies Anti-B. bovis TCTP in Rabbits

The immunization protocol and animal care were approved by the Bioethics Committee of the Faculty of Natural Sciences of the Autonomous University of Queretaro (15FCN2019). The trials were carried out at the facilities of the Faculty of Natural Sciences-UAQ. The selected peptides were synthesized by Peptide 2.0 using a MAPS 8 format, where 8 peptides bind to a lysine core, giving the molecule a larger size with a higher probability of producing higher antibody [26]. The synthesized peptides were solubilized individually in PBS and then prepared in a suspension containing 30% of the peptide diluted in PBS and 70% of the adjuvant Montanide ISA 71 VG (Seppic, Courbevoie, France) [27]. Each peptide was emulsified until homogeneous micelles were observed under the microscope (100×) and stored at 4 °C until use. All four peptides (I to IV) were used to immunize ten 6-week-old New Zealand rabbits individually in order to evaluate their immunogenicity and generate specific antisera. Animals were randomly assigned to groups. Before each immunization, blood was collected from the marginal ear artery. The blood was centrifuged at 2500 rpm for 300 s, and the serum was separated and frozen at −20 °C for further testing. For each peptide, two rabbits were immunized with 1 mL of the prepared immunogen (100 μg/mL) divided into two subcutaneous injections of 0.5 mL each (around the prescapular lymph nodes and around the inguinal lymph nodes); in addition, two rabbits were immunized with adjuvant and PBS as control. Rabbits were immunized 5 times every 21 days and euthanized for blood collection at the end of the study.

### 2.9. Immunization of Cattle with TCTP Peptides and Challenge with Virulent B. bovis

Male Holstein crossbred steers with previous negative results for the presence of antibodies against tuberculosis, *Brucella*, *B. bovis* and *B. bigemina* were divided into two groups of four animals each. One group of cattle was immunized with 60 µg of a mixture of peptides II, III and IV in PBS and Montanide ISA 71 VG adjuvant. A control group was immunized only with PBS and adjuvant. Animals were randomly assigned to groups. Peptide I was excluded from the mixture because its sequence overlaps with that of peptide IV. Peptide IV was specifically designed to include the predicted alpha-helix region, which distinguishes Apicomplexan TCTPs from mammalian TCTPs. The inclusion of both peptides was consequently considered redundant, and peptide IV was prioritized for the immunization protocol. Cattle were immunized with the peptide mixture three times with doses of 1 mL subcutaneously, every three weeks. Twenty-two days after the third immunization, both groups were challenged with 1 × 10^8^ erythrocytes infected with *B. bovis*. Clinical signs and parasitemia were monitored daily for 15 days. Parasitemia was measured daily by counting the total number of parasitized erythrocytes per 1000 erythrocytes in a Diff Quick (Hycel, Zapopan, Jalisco, México) stained blood smear, and clinical signs were recorded: rectal temperature, breathing rate, heart rate, capillary refill time, mucous membrane color, packed cell volume, ruminal movement and behavior. When steers developed severe clinical signs of babesiosis (presence of parasites in blood smears, more than 3 days with body temperature > 40.5 °C, decrease in packed cell volume (>40%) and signs such as anorexia, depression and lethargy), they were considered experimentally dead, defined as a clinical condition indicating a high probability of death if left untreated, and were treated with imidocarb diopropionate (1 mL/100 kg) and flunixin meglunin (1.1 mg/kg) to prevent death [28]. Serum samples were collected from all animals before each immunization and at three key time points: pre-immunization (day 0, immediately before the first immunization), pre-challenge (day 63, twenty-one days after the third immunization and before challenge), and post-challenge (day 79, sixteen days after challenge). Assays were performed at the Faculty of Natural Sciences-UAQ.

### 2.10. Confocal Microscopy

To determine if intraerythrocytic stages express TCTP, a confocal microscopy analysis was performed. Smears of *B. bovis*-infected red blood cells cultured in vitro were dried and stored at −80 °C until used. Then, they were vacuum-dried and permeated with 50% acetone and 50% methanol for 600 s at −80 °C. The slides were blocked with normal goat serum diluted (10%) in PBS with 0.05% Tween-20 (PBS-T). Then, they were incubated for 30 min at room temperature. The rabbit antisera were diluted 1:20 in PBS-T, added to the smears and incubated for 30 min at 37 °C in a humidity chamber. The slides were washed 3 times in PBS-T. A goat anti-rabbit IgG coupled with Alexa-488 (Thermo Scientific, Waltham, MA, USA) diluted 1:200 in PBS-T was used as a secondary antibody. Hoechst 33342 (Thermo Scientific, Waltham, MA, USA), and the smears were incubated for 30 min at 37 °C. Three washes were performed with PBS-T, and the last rinsing was performed with distilled water. Serum from a rabbit immunized with PBS and same adjuvant was used as a negative control. The slides were analyzed in a confocal microscope, with lasers for Hoechst 33342 and Alexa-488. A final merged imaged was composed using a bright field image as the third component with the LAS Advanced Fluorescence software 3.0 (Leica, Wetzlar, Germany).

### 2.11. Assessment of Specific Antibody Response to TCTP and Antibody Titration of Sera in Immunized Cattle

The generation of specific antibodies against BboTCTP was assessed by indirect ELISA using sera obtained from cattle on days 0, 21, 42 and 63 after the first immunization. For this, 96-well ELISA plates, Costar 3590, Corning (Corning, NY, USA) were sensitized with 4 µg/mL of peptides II, III and IV (100 µL/well) diluted in bicarbonate buffer. The plates were incubated at 4 °C overnight and washed 3 times with PBS-T 0.05%. The wells were blocked with 5% skim milk (Svelty®, Nestlé Japan Ltd., Tokyo, Japan), incubated at 37 °C for 1 h at 200 rpm and washed 3 times with PBS-T 0.05%. One hundred microliters of serum diluted 1:4000 from each sampling day (0, 21, 42 and 63 post immunization) was added. The plates were incubated at 37 °C for 1 h at 200 rpm and washed 3 times. One hundred microliters per well of peroxidase-conjugated affiniPure goat anti-bovine++ IgG (H + L), Jackson ImmunoResearch (West Grove, PA, USA) in a 1:6000 dilutions were added and incubated at 37 °C for 1 h at 200 rpm, and the washes were repeated. OPD substrate (Sigma-Aldrich, St. Louis, MO, USA) was added and allowed to incubate for 20 min, and the optical density (O.D.) was measured at 450 nm. Differences between sera were analyzed using Student’s *t*-test.

To determine antibody titers against BboTCTP present in the sera of immunized cattle, indirect ELISA was used. Briefly, ELISA plates were coated overnight at 4 °C with 4 µg/mL of each peptide (100 µL per well) and the plates were washed 3 times with PBS containing 0.05% Tween-20. After that, the plates were blocked with PBS-T 0.05% containing 5% of skim milk (Svelty®, Nestlé Japan Ltd., Tokyo, Japan) for 1 h at 37 °C and 200 rpm, followed by another 3 washes. The plates were incubated with 100 µL of sera collected on days 0 and 63 after the first immunization, serially diluted from 1:500 to 1:240,000. The plates were incubated at 37 °C for 1 h and 200 rpm, and then they were washed 3 times. A peroxidase-conjugated goat anti-bovine IgG (H + L) (Jackson Immunoresearch, West Grove, PA, USA), diluted 1:6000, was added (100 µL per well) and incubated for 1 h at 37 °C and 200 rpm. Three final washes were performed, and OPD substrate (Sigma-Aldrich, St. Louis, MO, USA) was added and incubated for 25 min. Optical density (OD) was measured at 450 nm. Differences between the two groups were analyzed using Student’s *t*-test, with significant differences set at (*p* < 0.05).

### 2.12. Determination of Total Antibodies in Bovine Sera

To test the hypothesis that antibodies against *B. bovis* TCTP block the effect of *B. bovis* TCTP to decrease the humoral immune response of cattle, the total circulating amount of serum antibodies was determined. For this, a titration of total antibodies was carried out in the bovine serum, using pre-immunization sera (day 0), the sera from the 3rd immunization (pre-challenge, day 63) and the sera taken 20 days after the challenge (day 79). Costar^®^ 96-well ELISA plates (ref. 3590) were sensitized with 100 µL of sera from each immunized animal at a dilution of 1:100 in carbonate buffer in triplicate and serially diluted from base 10 to 1:100,000,000,000. The plates were incubated overnight at 4 °C and washed 3 times with PBS-T 0.05%. They were then blocked with 5% skim milk (Svelty®, Nestlé Japan Ltd., Tokyo, Japan), incubated at 37 °C for 1 h at 200 rpm and washed again. A total of 100 µL per well of anti-bovine IgG (peroxidase-conjugated affiniPure goat anti-bovine++ IgG (H + L), Jackson Immunoresearch, Baltimore, MD, USA), at a 1:6000 dilution, was added and incubated at 37 °C for 1 h at 200 rpm and washed. OPD substrate (Sigma-Aldrich, St. Louis, MO, USA) was added, incubated for 25 min, and the optical density (OD) was measured at 450 nm. Statistically significant differences in OD (*p <* 0.05) between sera were analyzed by Student’s *t*-test comparing optical densities between the three serum uptakes.

### 2.13. Neutralization Assay

To assess whether antibodies against TCTP had a blocking effect on the erythrocyte invasion process, a neutralization assay was conducted using an in vitro culture of *B. bovis*. *B. bovis* was cultured using a 96-well plate, with each well containing 200 µL and a 5% hematocrit. Sera were filtered through a 0.22 µm membrane. Subsequently, they were heated at 56 °C for 30 min. Pre-incubation occurred in a 5% CO_2_, 5% O_2_ atmosphere at 37 °C for 30 min, in a culture medium containing 60% media, 40% sera, 1% iRBC and 1% parasitemia. Afterwards, 4% nRBCs were added, followed by gentle mixing, and the mixture was distributed into three wells. Cultures were maintained at 37 °C in a 5% CO_2_ and 5% O_2_ atmosphere for 72 h, with daily media changes (total volume 150 µL, consisting of 80% GIT media and 20% sera sample material). Controls included post-immunization serum from a steer immunized with PBS plus adjuvant, pre-immunization sera and GIT medium without serum. Parasitized erythrocytes were quantified by counting 2000 cells on Giemsa-stained slides at the end of the incubation period. Statistical analysis utilized ANOVA followed by a Tukey test, with significance set at *p* < 0.05, and were conducted using GraphPad Prism 9 software (San Diego, CA, USA).

## 3. Results

### 3.1. TCTP Is a Highly Conserved Gene in Babesia bovis

**Figure 1:**
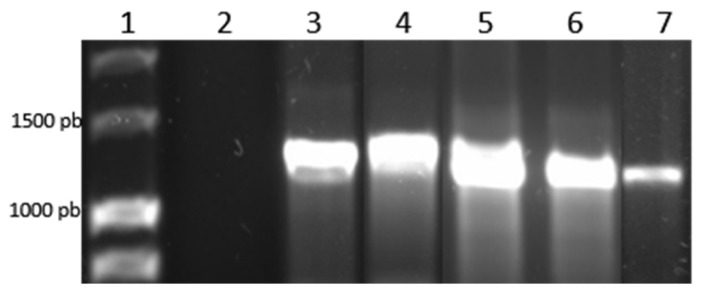
Amplification of the *tctp* gene in different *B. bovis* isolates: Lane 1: molecular size marker (1 kb); Lane 2: negative control; Lane 3: Michoacán-México isolate; Lane 4: Reference strain T2Bo; Lane 5: Jalisco-México isolate; Lane 6: Nayarit-México isolate; Lane 7: Zacatecas-México isolate. Expected band size 1199 bp.

**Figure 2:**
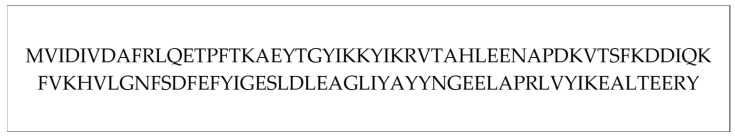
*B. bovis* TCTP consensus predicted amino acid sequence.

Amino acid sequence alignment of TCTP from *B. bovis* and *P. falciparum* revealed 93% protein coverage and 58% identity. Using bioinformatics, the nucleotide sequence was identified within the *B. bovis* genome, located on chromosome four, consisting of 889 bp with three exons and two introns. It is available in the NCBI database with Gene Id: 5478636 (U.S. National Library of Medicine, 2020). According to the PCR analysis, fragments of an expected size of 1119 bp were amplified in *B. bovis* isolates from Nayarit-México, Michoacán-México, Zacatecas-México and Puebla-México, as well as the reference strain T2Bo. For cloning, *tctp* gene cloned sequences were obtained from *B. bovis* isolates from Nayarit-México, Michoacan-México, Zacatecas-México and the reference strain T2Bo (Figure 1). With the sequences obtained from the different isolates, an alignment was made using the bioinformatics tool BLAST, with the sequence of the *tctp* gene reported in the NCBI, observing a nucleotide identity percentage of 99.2% in the *tctp* sequence of *B. bovis* isolate from Zacatecas-México, 99.6% % in the *tctp* sequence of *B. bovis* isolate from Michoacán-México and 100% identity at the amino acid level, thus obtaining a consensus sequence (Figure 2).

**Figure 3:**
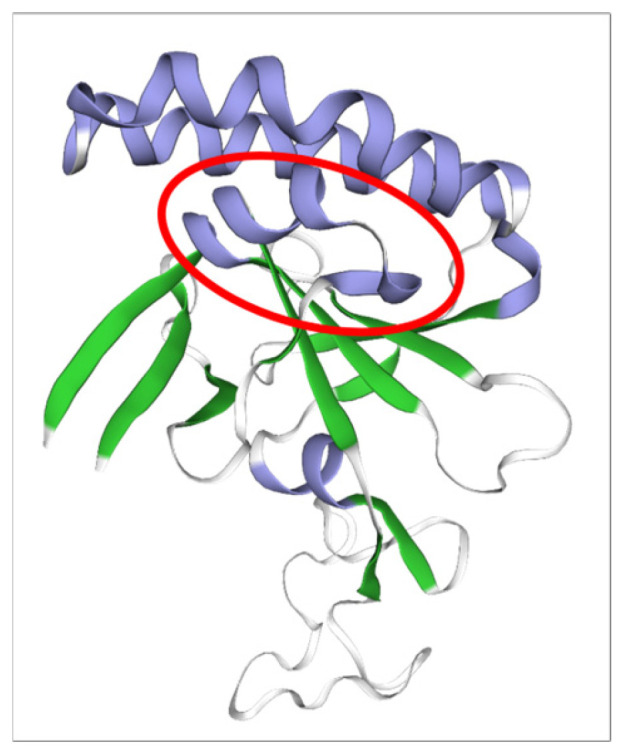
Bioinformatic prediction of the three-dimensional structure of *B. bovis* TCTP from amino acid sequence. Alpha-helix are shown in purple and beta-sheets are shown in green. The red circle indicates an alpha helix corresponding to amino acids 23–31, a region that is relevant to apicomplexan TCTPs.

In the topology analysis using bioinformatics tools, neither a signal peptide nor a transmembrane domain could be identified. However, a TCTP domain was identified. Multiple alignment of TCTP revealed 31.76% amino acid sequence identity between *Bos* spp. and *B. bovis* TCTP. In the multiple alignment between *B. bovis* TCTP and *B. bigemina* TCTP, 86.29% amino acid sequence identity was observed. Using bioinformatics modeling, the three-dimensional structure of TCTP was predicted from the sequence obtained from the sequenced isolates, with a tertiary structure very similar to that described in *P. falciparum* (3p3k.1.A). The alpha-helix corresponding to amino acids 23–31, described as rel-evant in apicomplexan TCTPs, was specifically annotated and highlighted in Figure 3.

### 3.2. Identification of B-Cell Epitope Candidates from BboTCTP

After bioinformatic analysis, four peptides ranging from 18 to 24 amino acids were selected and named TCTP peptides I to IV, whose detailed characteristics are shown in Table 1. Although only peptide III had an antigenicity score above the 0.4 threshold, the peptides were selected based on their location within conserved regions of the *B. bovis* TCTP sequence, their predicted B-cell epitopes, and their structural accessibility. These factors suggested that the peptides could be recognized by the immune system even if their antigenicity scores were below the standard threshold. Peptides II and III had predicted allergenicity scores above 0.4. However, it is important to note that these scores are in silico predictions, and their clinical significance has yet to be validated. No adverse effects were observed during immunization in rabbits or cattle, supporting their experimental safety and validating their use in the study.

### 3.3. TCTP Is Expressed in Intraerythrocytic Stages

**Figure 4:**
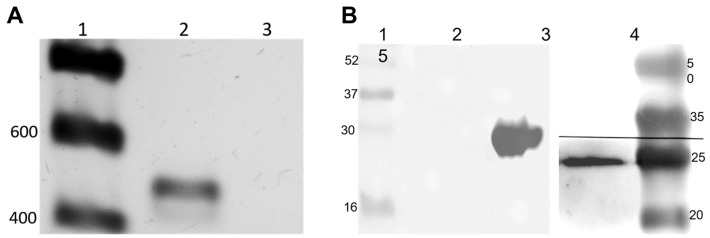
*Babesia bovis* TCTP is expressed by the erythrocytic parasite stages. (**A**) RT-PCR was visualized on agarose gel. Lane 1: DNA ladder marker; Lane 2: *B. bovis tctp* amplification (RT+); Lane 3: *B. bovis* mRNA (RT-). (**B**) Western blot showing a specific anti-TCTP antiserum reactivity. Lane 1. Prestained protein ladder shown in kiloDaltons (Opti-Protein Ultra Marker). Lane 2. Total extracts of non-infected bovine erythrocytes. Lane 3. Recombinant *B. bovis*-TCTP. Lane 4. Total extracts of infected bovine erythrocytes with *B. bovis.* Lane 5. Prestained protein ladder shown in kiloDaltons (Mid Range Protein Molecular Weight Marker).

To evaluate the expression of TCTP, the erythrocytic stages of *B. bovis* were analyzed for mRNA transcription. As observed in Figure 4, cDNA of *B. bovis*-infected erythrocytes was amplified by PCR, showing a band of expected size (426 bp) in agarose gel electrophoresis (Figure 4A, lane 2). No amplification was observed when the same mRNA sample was amplified without reverse transcriptase, confirming that the *tctp* gene is transcribed (Figure 4A, lane 3). Erythrocytic stages were analyzed for protein expression by Western blot (Figure 4B). For this, a rBboTCTP antiserum identified a specific band with a molecular weight of approximately 30 kDa (Figure 4B, lane 2). No signal was observed with the same antiserum incubated with proteins from uninfected red blood cells (Figure 4B, lane 3). The presence of the protein was confirmed by incubating recombinant BboTCTP protein with serum against rBboTCTP (Figure 4B, lane 4).

**Figure 5:**
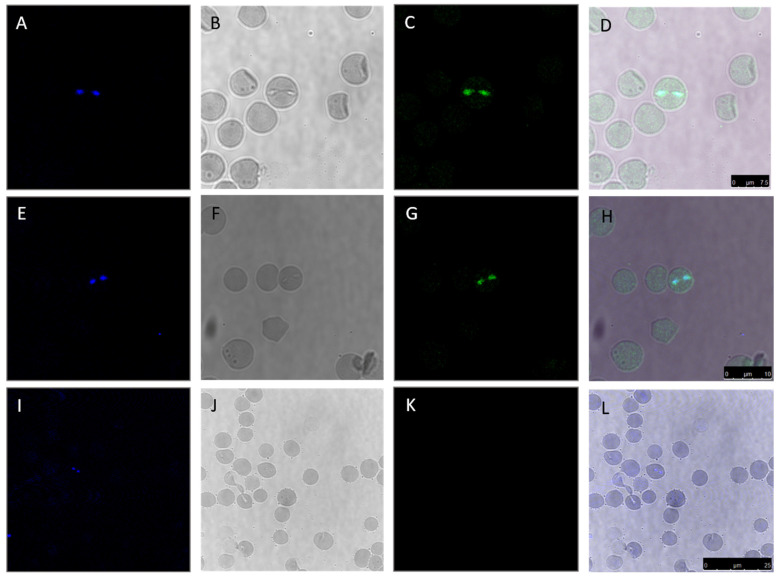
TCTP expression by *B. bovis* merozoites. Intraerythrocytic parasites were incubated with rabbit antiserum against peptide II (**C**,**D**) or rabbit antiserum against peptide IV (**G**,**H**). No signal was observed when merozoites with control serum from rabbit (**K**,**L**). Nuclei were stained with Hoechst 33342 (**A**,**E**,**I**). Bright field images (**B**,**F**,**J**) were also used to obtain merged images (**D**,**H**,**L**).

Rabbit sera immunized with individual TCTP peptides from *B. bovis* were evaluated by confocal microscopy to determine the expression of TCTP in intraerythrocytic stages of *B. bovis* (Figure 5). Using a rabbit antiserum for each peptide, merozoites were recognized by the respective antiserum (panels C and G). In contrast, no signal was detected when the parasites were incubated with control serum (panels K and L). As shown, TCTP has a cytoplasmic distribution (Figure 5, panels C, D, G, H). Together, these results confirm that TCTP is expressed in *B. bovis*, and the antibodies against BboTCTP peptides specifically recognize the native protein in intraerythrocytic stages.

### 3.4. BboTCTP Interferes with the Host Immune Response to Infection

**Figure 6:**
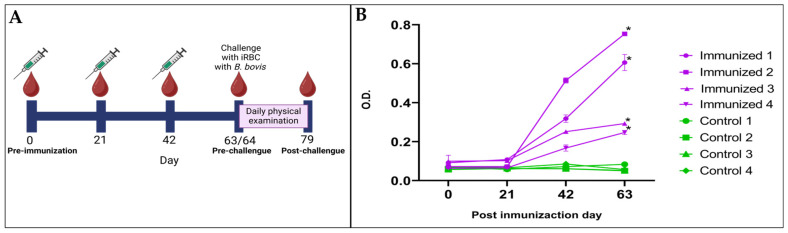
Immunization schedule and specific antibody response to BboTCTP in cattle. (**A**) Timeline showing the immunization and sampling schedule in cattle. Animals were immunized on days 0, 21, and 42 and challenged with B. bovis-infected erythrocytes on day 63. Serum samples were collected on days 0 (pre-immunization), 21, 42, 63 (pre-challenge) and 79 (post-challenge). (**B**) Graph of the determination of specific antibodies against BboTCTP on days 0, 21, 42 and 63. Immunized group in purple, control group in green. The asterisk indicates a statistically significant difference (*p* < 0.05) between pre-immunization and day 63 post-immunization serum.

After immunization, bovine serum obtained by indirect ELISA was analyzed to determine the reactivity of antibodies against the peptide mixture of BboTCTP. A difference (*p* < 0.05) in the amount of specific IgG antibodies against the peptide mixture was observed over the days (Figure 6). In the immunized group, specific antibodies against BboTCTP peptides increased from day 21 post-immunization and were detectable until day 63, when the challenge was performed. In contrast, the presence of specific antibodies against BboTCTP was not observed in the control group, which was immunized with adjuvant only.

After the challenge, a daily physical examination of the cattle was performed, and the parameters were measured and recorded daily for 15 days. The records of the animals were averaged and the standard deviation per group was taken, and the statistically significant difference was verified by Student’s *t*-test. In all animals, parasites were observed in the blood smear, and most of them had a temperature above 40.5 °C for 3 days; however, animals in the control group showed lethargy, depression, and anorexia. In the control group, experimental death was observed in three of the four animals treated to prevent death, whereas no animals in the vaccinated group required treatment. Less severe clinical signs were also observed in animals in this group. These clinical signs were confirmed by a blinded veterinarian (Table 2).

**Figure 7:**
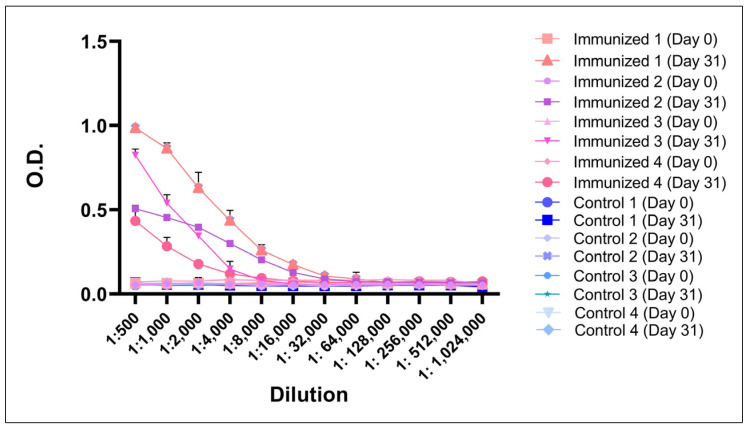
Specific IgG antibody titers to the BboTCTP peptides detected in the serum of cattle 31 days after immunization and the control group. The results are expressed as the mean ± SD.

In addition, it was observed that the level of specific antibodies against BboTCTP peptides in the serum of the animals on day 63 after immunization was also statistically different (*p* < 0.05) from the level of antibodies detected in the pre-immunization sera. As shown in Figure 7, antibody titers of up to 1:32,000 were observed 63 days after immunization. None of the pre-immunized or adjuvant-only immunized bovine sera contained antibody titers.

### 3.5. BboTCTP Interferes with the Immune Response of Cattle

**Figure 8:**
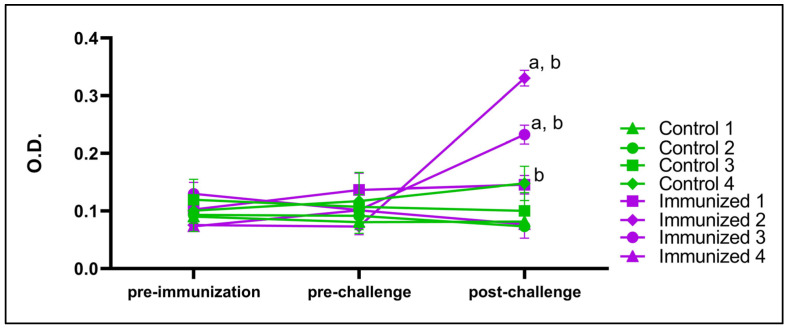
Graph of total antibodies in pre-immunization, pre-challenge and post-challenge sera. Immunized group in purple, control group in blue. a, statistical difference between pre-immunization and post-challenge serum (*p* < 0.05). b, statistical difference between pre- and post-challenge serum (*p* < 0.05).

The bovine serum was analyzed by indirect ELISA to evaluate the total antibodies to verify if there was a difference in the level of total antibodies between the animals in the control and vaccinated groups. The titration of total antibodies in bovine serum was performed with the pre-immunization cattle sera, the sera from the pre-challenge (day 63) and the sera post challenge (day 79). In the control group, it was observed that in three of the four animals (Controls 1, 2 and 3), the absorbance measured in the post-challenge serum was the same as in the pre-immunization (day 0) and pre-challenge (day 63) serum. In the immunized group, it was observed that in three of the four animals (Immunized 1, 2 and 3), the absorbance measured in the post-challenge (day 79) serum was higher than in the pre-immunization (day 0) and pre-challenge sera (day 63) (Figure 8).

### 3.6. Antibodies Anti rBboTCTP Block Invasion of Merozoites to Erythrocytes In Vitro

**Figure 9:**
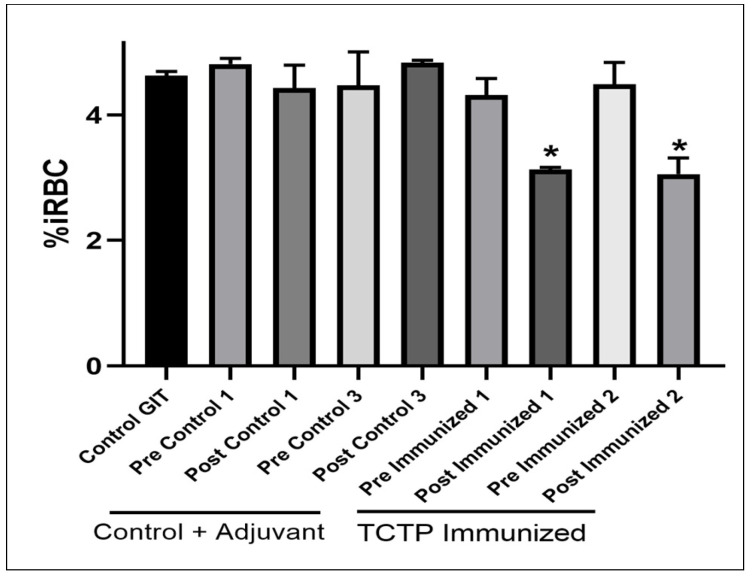
Neutralization assay against *B. bovis*. Post-immunization sera from cattle immunized with *B. bovis* TCTP resulted in a decrease in the number of infected erythrocytes compared to the control group (GIT Control). The graph displays pre- and post-immunization sera from controls administered only adjuvant, as well as pre- and post-immunization sera with TCTP. The mean results (iRBC%) and standard deviation are shown. ANOVA with Tukey’s test was performed. * Statistically significant differences are indicated by an asterisk (*p* < 0.05).

Antisera anti-TCTP antibodies exhibited a slight neutralizing effect by reducing the quantity of iRBCs at the time of evaluation compared to the controls (Figure 9). In the control containing only GIT medium, the mean was 4.62% iRBCs. For the pre-immunization controls, the following results were obtained: 4.81%, 4.47%, 4.32% and 4.48%. In the case of post-immunization sera, controls with adjuvant showed parasitic growth of 4.43% and 4.83%, respectively; none of these exhibited statistically significant differences compared to the control. The two sera with anti-TCTP antibodies showed 3.13% and 3.05% iRBCs, resulting in a decrease of 32.29% and 33.88% (*p* < 0.05) in the quantity of infected cells, respectively.

## 4. Discussion

The identification and characterization of TCTP in *B. bovis* is important because of its potential role in the host immune response, and its potential use as a vaccine is being studied in other organisms of the phylum Apicomplexa. This is the first study characterizing TCTP in *Babesia bovis*. Our results showed that BboTCTP is expressed during the intraerythrocytic phase, contains conserved B epitopes that are immunogenic and is potentially involved in modulating the host immune response. These results provide new insights into the biology of *B. bovis* and open new perspectives for the development of specific immunoprophylactic strategies.

### 4.1. TCTP Is a Highly Conserved

In this study, we observed that *B. bovis* TCTP is highly conserved among different isolates geographically, similar to what has been observed for TCTP in different isolates of *Plasmodium yoelii* [17]. It was also observed that this protein is conserved among different species of the phylum Apicomplexa, being 58% similar to the protein in *P. falciparum* and 81% similar to the protein in *B. bigemina*. It was described that there are structural variations between *Plasmodium* TCTP and human TCTP, particularly in the domain spanning residues 22–30 near the NH2 end, which in human TCTP forms a beta-sheet, whereas in *Plasmodium* spp. TCTP there is an alpha helix. We observed the same alpha helix between amino acids 23 and 31 in BboTCTP, and we also observed that this protein also lacks a signal peptide, similar to that described in TCTP from other species [16]. This high degree of conservation reinforces its potential as a broadly applicable immunogen in all *Babesia* species.

### 4.2. BboTCTP Expression During the Erythrocytic Stage

Transcription and expression of the *B. bovis tctp* gene were analyzed. The transcription of *tctp* was detected by RT-PCR and protein expression was analyzed by Western blot, both in intraerythrocytic stages of the parasite. The confirmed transcription and translation of *tctp* supports its role as an active gene product during the erythrocytic stage and enhances its biological relevance as a target. In addition, peptide-specific antibodies were generated, which recognized the native antigen as evaluated by confocal microscopy, with a cytoplasmic distribution consistent with reports in other species [9,29]. These results confirm that TCTP is a protein expressed in intraerythrocytic stages of *B. bovis*, which is consistent with what occurs with other Apicomplexa organisms.

### 4.3. Potential Inhibitory Effect of Anti-TCTP Antibodies on Parasite Invasion

To evaluate the ability of anti-BboTCTP antibodies to block erythrocyte invasion, an in vitro neutralization assay was performed. Percent inhibition was observed using serum generated against the peptide mixture. The fact that bovine sera immunized against *B. bovis* TCTP exhibited a neutralizing effect, resulting in a decrease in the number of infected erythrocytes detected compared to the control, may be attributed to the following factors: first, TCTP peptides may play a key role in the host immune response by inducing the production of specific antibodies that neutralize the activity of the erythrocytic phases of the parasite in vitro. On the other hand, TCTP could directly interfere with the cell invasion mechanisms of *B. bovis* or its ability to adhere to erythrocytes. In addition, TCTP could modulate the expression of genes related to the virulence of the parasite, resulting in a reduction of its infectious capacity. In *Toxoplasma gondii*, a higher level of expression has been described in tachyzoites of strains with higher virulence [30]. Although the detailed mechanism remains to be elucidated. These observations raise the possibility that TCTP plays a functional role in invasion or virulence.

### 4.4. Protective Effects of TCTP Immunization in Challenged Cattle

This protein has previously been tested as a vaccine candidate against *Plasmodium* spp. and caused a reduction in parasitemia and a delay in peak parasitemia in vaccinated mice after challenge. These effects were attributed to interference with events necessary for the establishment of infection, such as histamine release, basophil activation and inhibition of B and T cell activation [24]. In our challenge with the field isolate of *B. bovis*, three of the four animals in the adjuvant-immunized control group required treatment to prevent death, with less severity of clinical signs and faster recovery observed in cattle in the immunized group. A study was conducted in which mice were immunized three times (every 8 weeks) with *Plasmodium falciparum* or *P. yoelii* TCTP and challenged 6 weeks later with different strains of *P. yoelii*; a decrease in parasitemia and a delay in the appearance of infected erythrocytes were also observed in immunized mice compared to mice in the control group immunized with PBS [31]. It was mentioned that in mice inoculated with *P. berguei* without the *tctp* gene, a reduction and delay in the development of parasitemia was observed, in addition to a 90% reduction in neurological signs compared to the control group. It has also been reported that when mice are immunized with *P. falciparum* TCTP or *P. yoelii* TCTP and challenged with *P. yoelii*, a reduction and delay in the onset of parasitemia is observed [9]. Overall, these observations are consistent with previous studies in *Plasmodium* and support the hypothesis that TCTP-based immunization may confer protection by interfering with early infection.

### 4.5. Antibody Levels and Possible Immunomodulatory Role of BboTCT

In terms of total antibody titers, we observed similar levels in the control group at all three sampling intervals, with slightly lower titers in post-challenge sera compared to pre-immunization samples. In contrast, the immunized group showed elevated total antibody levels in the post-challenge sera. This difference could be related to the specific antibody response to *B. bovis* TCTP (especially because animals with higher anti-TCTP antibody titers showed greater differences between sampling points), in the context of the hypothesis that TCTP inhibits the humoral immune response by preventing the activation of antibody-secreting plasma cells, as proposed in other studies [13].

These results highlight the importance of TCTP as a potential target for the development of strategies to control and prevent bovine babesiosis. However, our current study does not include functional assays to support this mechanism. Therefore, further studies are needed to investigate the potential immunomodulatory role of *B. bovis* TCTP and its interaction with bovine B cells. This is the first study to characterize TCTP in *Babesia bovis*, a protein previously studied in other apicomplexan parasites such as *Plasmodium*. Our results demonstrate that BboTCTP is expressed during the intraerythrocytic phase, contains conserved B epitopes that are immunogenic, and may be involved in modulating the host immune response. These results provide new insights into the biology of *B. bovis* and open new perspectives for the development of specific immunoprophylactic strategies.

## 5. Conclusions

In this study, we provide the first experimental characterization of TCTP in *Babesia bovis*. BboTCTP was shown to be highly conserved among isolates, expressed during the intraerythrocytic phase, and to contain B-cell epitopes capable of inducing a specific antibody response in both rabbits and cattle. Immunized animals developed a strong antibody response and partial protection was observed after challenge, supporting the potential of BboTCTP as an immunogenic target.

These findings provide new insights into the biology and immunological relevance of *B. bovis* and suggest that TCTP may play a role in immune modulation and parasite survival. However, further studies are needed to elucidate the precise mechanisms by which TCTP affects host immunity, particularly in relation to B cell function and antibody production. Future work should also explore the use of TCTP in combination with other antigens to enhance protective efficacy and evaluate its potential application in diagnostic tools or vaccines.

## Figures and Tables

**Table 1:** Characteristics of the peptides selected for synthesis, with size and location within the amino acid sequence of the *B. bovis tctp* gene.

Peptide	Sequence	Amino Acid	Position	Antigenicity Value	Allergenicity Value
Peptide TCTP I	GDEVCSDAYTHLNPFDNPE	19	10–28	0.3626 (non-predicted)	0.1147 (predicted)
Peptide TCTP II	TGYIKKYIKRVTAHLEENAPD	21	92–112	−0.1684 (non-predicted)	0.3931 (predicted)
Peptide TCTP III	SSKVAKGNEDYGIACNDDEEGGG	23	38–60	1.6397 (predicted)	0.1443 (predicted)
Peptide TCTP IV	CSDAYTHLNPFDNPEFASVAFE	22	14–35	0.3216 (non-predicted)	0.2153 (predicted)

Antigenicity values were obtained from the VaxiJen v2.0 server (https://www.ddg-pharmfac.net/vaxijen/, accessed on 19 November 2024), with values above the 0.4 threshold considered probable antigens. Allergenicity values were obtained from the AlgPred 2.0 server (https://webs.iiitd.edu.in/raghava/algpred/, accessed on 19 November 2024), with values under the 0.4 threshold considered probable allergens.

**Table 2:** Clinical signs observed in each animal to determine experimental death and provide treatment.

	Maximum Parasitemia in Peripheral Blood	Temperature > 40.5 °C More Than 3 Days	Decrease in Mean Corpuscular Volume > 40%	Lethargy, Depression	Anorexia	Experimental Death
Control	0.05%	+	+	+	+	x
	0.45%	+		+	+	x
	0.45%	+	+			
	0.25%	+	+	+	+	x
Immunized	0.10%	+				
	0.35%		+			
	0.25%					
	0.20%	+	+			

## Data Availability

The original contributions presented in the study are included in the article; further inquiries can be directed to the corresponding authors.

## Abbreviations

The following abbreviations are used in this manuscript:

*B. bovis*	*Babesia bovis*
*B. bigemina*	*Babesia bigemina*
*B. divergens*	*Babesia divergens*
IL	Interleukin
BLAST	Basic Local Aligment Search Tool
DNA	Deoxyribunuclei chain reaction
PCR	Polymerase chain reaction
s	second
°C	Degrees Celcius
UNAM	National Autonomous University of Mexico
RT-PCR	Reverse transcription polymerase chain reaction
RNA	Ribonucleic Acid
µg	Micrograms
cDNA	Complementary DNA
µL	Microliter
bp	Base pairs
WB	Western blot
iRBC	Infected red blood cells
SDS	Sodium dodecyl sulfate
PBS	Phosphate buffered saline
mM	Milimolar
h	Hour
TBS	Tris-buffered Saline
MAPS	Multiple antigenic peptide
rpm	Revolution per minute
mL	Milliliter
OPD	O-phenylenediamine dihydrochloride
ELISA	Enzyme-liked immunosorbent assay
µm	Micrometer
nRBC	Non-infected red blood cells
ANOVA	Analysis of variance
NCBI	National Center for Biotechnology Information
mRNA	Messenger RNA
